# Supplementary material for: Balancing Speed and Coverage by Sequential Seeding in Complex Networks
Source: Sci Rep. 2017 Apr 18;7:891. doi: 10.1038/s41598-017-00937-8 (PMC5429852; doi:10.1038/s41598-017-00937-8)
Supplement: Supplementary file 1 — Supplementary Information [file 41598_2017_937_MOESM1_ESM.pdf]

Balancing Speed and Coverage by Sequential Seeding in Complex Networks

Jarosław Jankowski[1,2,*], Piotr Bródka[1], Przemysław Kazienko[1], Boleslaw K. Szymanski[1,3], Radosław Michalski[1], and Tomasz Kajdanowicz[1]

[1]Wrocław University of Science and Technology, Department of Computational Intelligence, Wrocław, 50-370, Poland
[2]West Pomeranian University of Technology, Department of Computer Science and Information Technology, Szczecin, 71-210, Poland
[3]Rensselaer Polytechnic Institute, Department of Computer Science, Troy, 12180, United States
[*]jjankowski@wi.zut.edu.pl

Supplementary information

Specification of networks used in simulations

An experimental setup runs agent-based simulations on 15 static real networks specified in Table 1.

Id	Network	Nodes	Edges	Reference
N1	Condensed matter collaborations 1999	16,726	47,594	1
N2	UC Irvine messages	1,899	59,835	2
N3	Political blogs	1,490	19,090	3
N4	Power Grid	4,941	6,594	4
N5	Scholarly Collaboration in Network Science	1,589	2,742	5
N6	ego-Facebook	4,039	88,234	6
N7	General Relativity and Quantum Cosmology collaboration network	5,242	14,496	7
N8	DBLP	12,591	49,743	8
N9	Hamsterster friendships	1,858	12,534	9
N10	UC Irvine forum	899	33,720	10
N11	University of Oregon Route Views	6,474	13,895	7
N12	Communication at the University Rovira i Virgili	1,133	5,451	11
N13	US airports	1,574	28,236	12
N14	JUNG and javax dependency	6,120	138,706	13
N15	Reactome project	6,327	147,547	14

Table 1. Networks used in simulations

Comparison of results with other seeding methods

For clarity the main part of manuscript contains comparison of sequential seeding approach with benchmark methods based on typical network structural measures. As we discussed in the introduction, the sequential seeding can be used to improve any single stage approach. Here we present comparison of sequential seeding to most recent seed selection/ranking methods designed to minimize the probability of choosing the nodes that might activate each other. Results from simulations with VoteRank[15] and community structure based[16] methods are presented in Fig. 1. Single stage (SN) and sequential (SQ) approaches were compared for both methods as well as with results obtained from sequential seeding based on degree ranking chosen as an example of benchmark method. For VoteRank method experimental space was based on 450 configurations (15 networks, 6 propagation probabilities and 5 seeding percentages). While all simulations presented in the paper were performed for five seeding percentages,

other approach was used for comparison with community structure based method because this method uses number of seeds related to the number of identified communities. Accordingly, the number of seeds for sequential seeding was set the same as for single stage seeding. Lacking the seeding percentage parameter the experimental space consisted of 90 configurations (15 networks and 6 propagation probabilities). Average coverage for degree based ranking sequential seeding (SQ_Degree) was 1.22 times higher than for VoteRank single stage approach (SN_VoteRank). Sequentially used seeds from VoteRank (SQ_VoteRank) delivered 1.25 times better coverage than used in single stage, see Fig. 1 A. The results for SQ_Degree delivered better results than SN_VoteRank in 84.89% of simulation configurations (Fig. 1 B). In 89.56% of simulation cases SQ_VoteRank delivered better results than SN_VoteRank (Fig. 1 C). SQ_Degree delivered on average 1.15 times higher coverage than single stage community structure based seeding (SN_Community) (Fig. 1 D). Sequential approach (SQ_Community) delivered average 0.47% increase of coverage when compared to SN_Community. Results from SQ_Degree delivered better results than SN_Community in 93.33% of simulation configurations (Fig. 1 E). In 66.67% of configurations SQ_Community produced better results than SN_Community (Fig. 1 F).

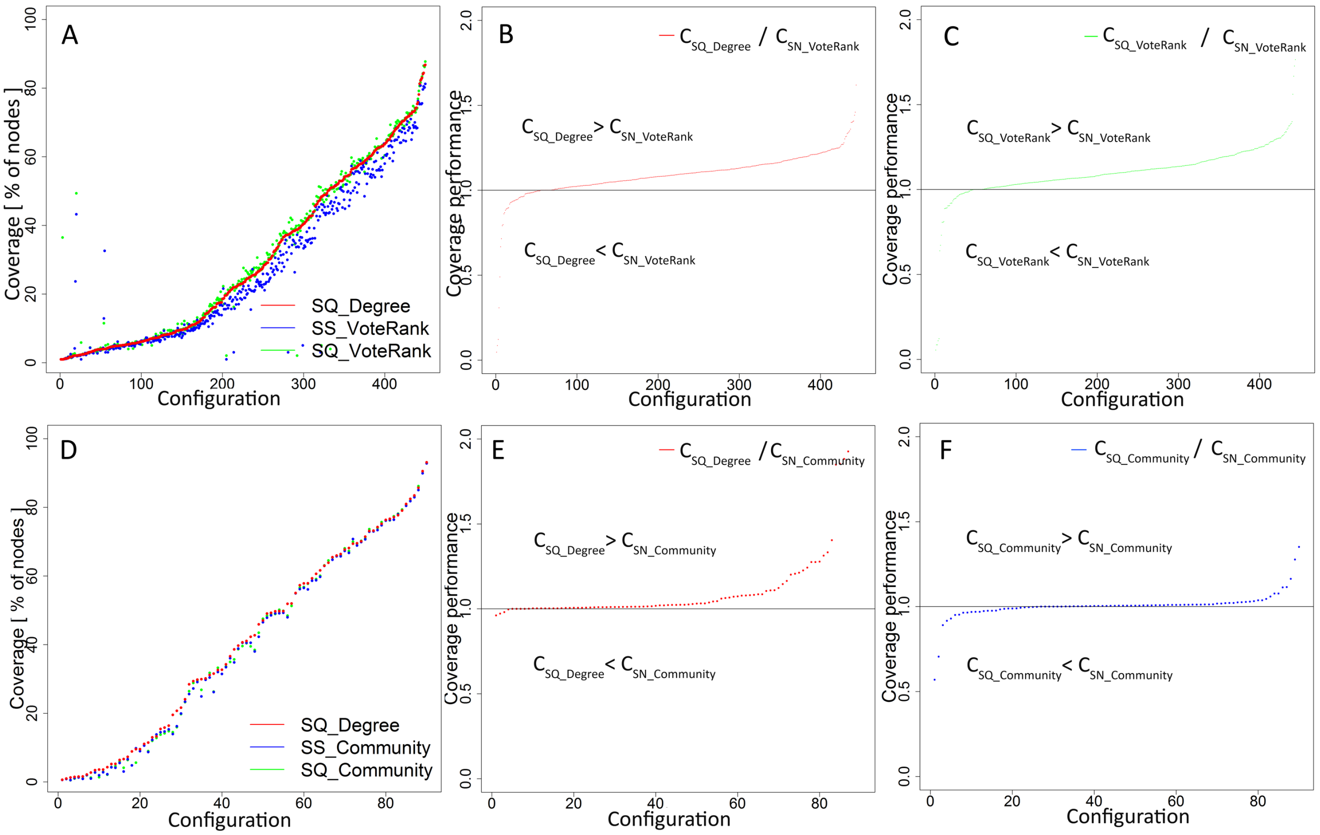

Figure 1. **(A)** Average coverage from 450 configurations for $k = 1$ sequential seeding with revival mode based on degree rankings (SQ_Degree) compared with results from process initiated by seeds computed with the VoteRank method used in single stage (SN_VoteRank) and in sequential seeding (SQ_VoteRank) for all simulation cases ordered by coverage obtained in SQ_Degree; **(B)** Performance of $k = 1$ per stage sequential seeding (SQ_Degree) in the relation to single stage seeding based on seeds from VoteRank (SN_VoteRank) for all configurations; **(C)** Performance of $k = 1$ per stage sequential seeding from VoteRank method (SQ_VoteRank) in the relation to single stage seeding VoteRank (SN_VoteRank) for all configurations; **(D)** Average coverage from 90 configurations for $k = 1$ sequential seeding with revival mode based on degree rankings (SQ_Degree) compared with results from process initiated by seeds computed by community structure based method used in single stage (SN_Community) and in sequential seeding (SQ_Community) ordered by coverage obtained in SQ_Degree; **(E)** Performance of $k = 1$ per stage sequential seeding (SQ_Degree) in the relation to single stage seeding SN_Community; **(F)** Performance of $k = 1$ per stage sequential seeding SQ_Community in the relation to a SN_Community for all configurations;

References

1. Newman, M. E. Scientific collaboration networks. i. network construction and fundamental results. *Physical review E* **64**, 016131 (2001).
2. Opsahl, T. & Panzarasa, P. Clustering in weighted networks. *Social networks* **31**, 155–163 (2009).
3. Adamic, L. A. & Glance, N. The political blogosphere and the 2004 us election: divided they blog. In *Proceedings of the 3rd international workshop on Link discovery*, 36–43 (ACM, 2005).
4. Watts, D. J. & Strogatz, S. H. Collective dynamics of 'small-world'networks. *nature* **393**, 440–442 (1998).
5. Newman, M. E. Finding community structure in networks using the eigenvectors of matrices. *Physical review E* **74**, 036104 (2006).
6. Leskovec, J. & Mcauley, J. J. Learning to discover social circles in ego networks. In *Advances in neural information processing systems*, 539–547 (2012).
7. Leskovec, J., Kleinberg, J. & Faloutsos, C. Graph evolution: Densification and shrinking diameters. *ACM Transactions on Knowledge Discovery from Data (TKDD)* **1**, 2 (2007).
8. Ley, M. The dblp computer science bibliography: Evolution, research issues, perspectives. In *International Symposium on String Processing and Information Retrieval*, 1–10 (Springer, 2002).
9. KONECT. Hamsterster friendships network dataset (2016).
10. Opsahl, T. Triadic closure in two-mode networks: Redefining the global and local clustering coefficients. *Social Networks* **35**, 159–167 (2013).
11. Guimera, R., Danon, L., Diaz-Guilera, A., Giralt, F. & Arenas, A. Self-similar community structure in a network of human interactions. *Physical review E* **68**, 065103 (2003).
12. Opsahl, T. Why anchorage is not (that) important: Binary ties and sample selection. *online] http://toreopsahl.com/2011/08/12/why-anchorage-is-not-that-important-binary-tiesand-sample-selection (accessed September 2013)* (2011).
13. Šubelj, L. & Bajec, M. Software systems through complex networks science: Review, analysis and applications. In *Proceedings of the First International Workshop on Software Mining*, 9–16 (ACM, 2012).
14. Joshi-Tope, G. *et al.* Reactome: a knowledgebase of biological pathways. *Nucleic acids research* **33**, D428–D432 (2005).
15. Zhang, J.-X., Duan-Bing Chen, Q. D. & Zhao, Z.-D. Identifying a set of influential spreaders in complex networks. *Scientific reports* **6** (2016).
16. He, J.-L., Fu, Y. & Chen, D.-B. A novel top-k strategy for influence maximization in complex networks with community structure. *PloS one* **10**, e0145283 (2015).
